# The role of hypoxia-related genes in TACE-refractory hepatocellular carcinoma: Exploration of prognosis, immunological characteristics and drug resistance based on onco-multi-OMICS approach

**DOI:** 10.3389/fphar.2022.1011033

**Published:** 2022-09-26

**Authors:** Xuelian Cheng, Jingjing Li, Limei Feng, Songwei Feng, Xiao Wu, Yongming Li

**Affiliations:** ^1^ School of Medicine and Holistic Integrative Medicine, Jiangsu Collaborative Innovation Center of Chinese Medicinal Resources Industrialization, Nanjing University of Chinese Medicine, Nanjing, China; ^2^ School of Medicine, Southeast University, Nanjing, China; ^3^ State Key Laboratory of Bioelectronics, School of Biological Science and Medical Engineering, Southeast University, Nanjing, China

**Keywords:** TACE, drug resistance, hepatocellular carcinoma, prognosis, hypoxia

## Abstract

Transcatheter arterial chemoembolization (TACE) is an effective treatment for hepatocellular carcinoma (HCC). During TACE, chemotherapeutic agents are locally infused into the tumor and simultaneously cause hypoxia in tumor cells. Importantly, the poor effect of TACE in some HCC patients has been shown to be related to dysregulated expression of hypoxia-related genes (HRGs). Therefore, we identified 33 HRGs associated with TACE (HRGTs) by differential analysis and characterized the mutational landscape of HRGTs. Among 586 HCC patients, two molecular subtypes reflecting survival status were identified by consistent clustering analysis based on 24 prognosis-associated HRGs. Comparing the transcriptomic difference of the above molecular subtypes, three molecular subtypes that could reflect changes in the immune microenvironment were then identified. Ultimately, four HRGTs (*CTSO*, *MMP1*, *SPP1*, *TPX2*) were identified based on machine learning approachs. Importantly, risk assessment can be performed for each patient by these genes. Based on the parameters of the risk model, we determined that high-risk patients have a more active immune microenvironment, indicating “hot tumor” status. And the Tumor Immune Dysfunction and Exclusion (TIDE), the Cancer Immunome Atlas (TCIA), and Genome of Drug Sensitivity in Cancer (GDSC) databases further demonstrated that high-risk patients have a positive response to immunotherapy and have lower IC50 values for drugs targeting cell cycle, PI3K/mTOR, WNT, and RTK related signaling pathways. Finally, single-cell level analysis revealed significant overexpression of *CTSO*, *MMP1*, *SPP1*, and *TPX2* in malignant cell after PD-L1/CTLA-4 treatment. In conclusion, Onco-Multi-OMICS analysis showed that HRGs are potential biomarkers for patients with refractory TACE, and it provides a novel immunological perspective for developing personalized therapies.

## Introduction

Hepatocellular carcinoma (HCC) accounts for 85% of all liver cancers (Villanueva., 2019). Despite advances in treatment strategies for HCC, the overall 5-years survival rate for patients with HCC remains below 20% (Zheng et al., 2018). Transcatheter arterial chemoembolization (TACE) is a therapy in which drugs are injected into the arteries supplying HCC tissue (Chang et al., 2020). Some studies suggest that TACE-refractory may lead to poor prognosis in HCC patients. It has been shown that TACE procedures can exacerbate hypoxic states (Liu et al., 2016). However, we still lack a multi-omics data-based perspective on the immunological characteristics of hypoxia-associated gene sets in TACE-refractory patients.

Hypoxia is an intrinsic feature of solid tumors due to the imbalance between tumor cell proliferation rate and vascular nutrient supply (Gray et al., 1953). Previous studies have shown that hypoxia can regulate tumor immune microenvironment (TME), such as promoting the recruitment of innate immune cells, and interfering with the differentiation and function of adaptive immune cells (Feng et al., 2022a). Certain cytokines secreted by malignant tumors, especially in hypoxia condition, may induce angiogenesis and metastasis (Zarogoulidis et al., 2014). A retrospective clinical study has shown that high pre-treatment IL-8 levels are a significant predictor of shorter survival and increased refractoriness of TACE (Kim et al., 2015). Therefore, further studies are needed to investigate the hypoxia-related genes (HRGs) that contribute to TACE refractoriness. Importantly, exploring the relationship between the above genes and drug resistance can lead to the development of new therapeutic strategies.

Nowadays, the study of molecular mechanisms based on Onco-Multi-OMICS approach has become one of the most important tools (Feng et al., 2022b; Zhu et al., 2022). Therefore, we searched for hub HRGs contributing to TACE refractoriness and searched for optimal biological markers by combining transcriptome, single cellome, immunome, and whole-exome. Our study also illustrated the immunological characteristics in different risk group and explored their impact on the response to chemotherapy and immunotherapy. In conclusion, our results were beneficial for the management and treatment of TACE-refractory patients.

## Materials and methods

### Data collection and pre-processing

The mRNA expression profile data of HCC patients were retrieved from TCGA and GEO databases, and the exclusion criteria was as follows: lack of complete follow-up information, 0 days of survival, and repeated sequencing. Supplementary Table S1 showed treatment details for patients in the GSE14520 cohort before exclusion. Finally, 365 tumor samples from the TCGA-LIHC cohort and 221 tumor samples from the GSE14520 cohort were included. Moreover, to study TACE response, we obtained gene expression profile data from GSE104580, which included 100 TACE-responsive samples and 100 TACE-refractory samples (He et al., 2022). Both somatic mutation data and CNV data were obtained from the TCGA-LIHC cohort, including 371 tumor samples. Notably, ‘maftools’ package was used to present the mutation status of each gene. We removed the batch effect between RNA-seq and microarray data by using the ‘sva’ package and made the newly generated gene matrix based on two cohorts as a meta cohort.

### Identification of hypoxia-related genes in TACE refractoriness

Differentially expressed genes (DEGs) between different response states were identified by using the ‘limma’ package in the GSE104580 cohort, *p* < 0.05, with |logFC| > 1 as the threshold. In addition, 1,694 HRGs were extracted from the previous study (Zhang et al., 2020). The above DEGs and HRGs were overlaid to identify the HRGs associated with TACE (HRGTs).

### Enrichment analysis

Differential genes between subtypes were analyzed using the ‘limma’ package (adj. *p* < 0.05, |logFC| > 1). Biological pathways were annotated by using the ‘clusterProfiler’ package for Gene Ontology (GO), Kyoto Gene and Genome Encyclopedia (KEGG). p-value < 0.05 and q-value < 0.05 were considered as significant enrichment pathways. Differences in biological pathways between subtypes were assessed by using ‘GSVA’ algorithm. And KEGG geneset (c2. cp. kegg. v7. 0. symbols. Gmt) was used as the reference gene set with FDR <0.05 as the threshold.

### Consistent clustering analysis

In the meta cohort, the prognostic value of each HRGTs was determined by using univariate cox regression analysis. Consensus clustering, an unsupervised clustering method, is a common method to classify subtypes based on the CDF slope was smallest. Consistent cluster analysis and principal component analysis (PCA) were performed to determine whether each subtype was relatively independent of the other subtypes based on prognostic HRGTs (*p* < 0.05) and prognostic DEGs (*p* < 0.05). The number of clusters was determined by using ‘conensusClusterPlus’ package. A 1000 replicates with pltem = 0.8 were performed to verify the stability of the subtypes. We used Kaplan Meier analysis and log-rank test to assess the overall survival (OS) of HCC patients in different subtypes.

### Identification and validation of risk scores

Modeling and validation were performed by TCGA-LIHC cohort and GSE14520 cohort, respectively. The least absolute shrinkage and selection operator (LASSO) (Feng et al., 2022c) model was used to remove redundant genes from HRGTs. Subsequently, multivariate Cox regression analysis was performed to integrate the coefficients and then establish risk score formulas by gene expression values. Univariate and multivariate Cox regression analyses were used to assess the prognostic value of risk scores across the entire dataset and the external validation dataset. Time-dependent subject operating characteristic (ROC) curves were used to compare the predictive accuracy of risk scores with traditional clinicopathological parameters.

### Drug sensitivity analysis

Half maximal inhibitory concentration (IC50) was calculated using the ‘prophetic’ package. Relevant drugs targeting cell cycle, PI3K/mTOR, WNT, and RTK pathways were obtained from the Genome of Drug Sensitivity in Cancer (GDSC) database. Charoentong et al. developed a quantitative scoring scheme called the Immunophenotype Score (IPS) to identify the determinants affecting tumor immunogenicity. IPS is a representative gene associated with immunogenicity calculated using z-score, and our meta cohort’s IPS was calculated from the Cancer Immunome Atlas (TCIA) database (Wu et al., 2018). Moreover, Peng et al. designed a computational architecture, Tumor Immune dysfunction and ejection (TIDE) score (Jiang et al., 2018), to integrate the two mechanisms of tumor immune escape. Our meta cohort’s TIDE score was calculated from the TIDE database.

### Single-cell analysis

The HCC single cell dataset was obtained from GSE125499, and single cell expression profile with annotated cell information were obtained from the Tumor Immune Single Cell Hub (TISCH) database (Sun et al., 2021). Finally, we compared the expression changes of *CTSO*, *MMP1*, *SPP1*, *TPX2* in different cell types.

### Immunological analysis

We used different algorithms to estimate the abundance of immune cells in different samples, such as ssGSEA, TIMER, CIBERSORT, QUANTISEQ, MCP-counter, XCELL and EPIC. Then, ESTIMATE algorithm was used to calculate the immune score and stromal score to reflect the TME status.

### Statistical analysis

All statistical analyses were performed using the R software (v.4.1.1). Kruskal-wallis test was used for comparison between groups, χ2 test was used for association between covariates, and Kaplan-Meier method was utilized to compare survival differences between groups. More detailed statistical methods for transcriptome data processing are covered in the above section (Ye et al., 2022). *p* < 0.05 was considered statistically significant.

## Results

### Landscape of HRGTs in HCC

**FIGURE 1:**
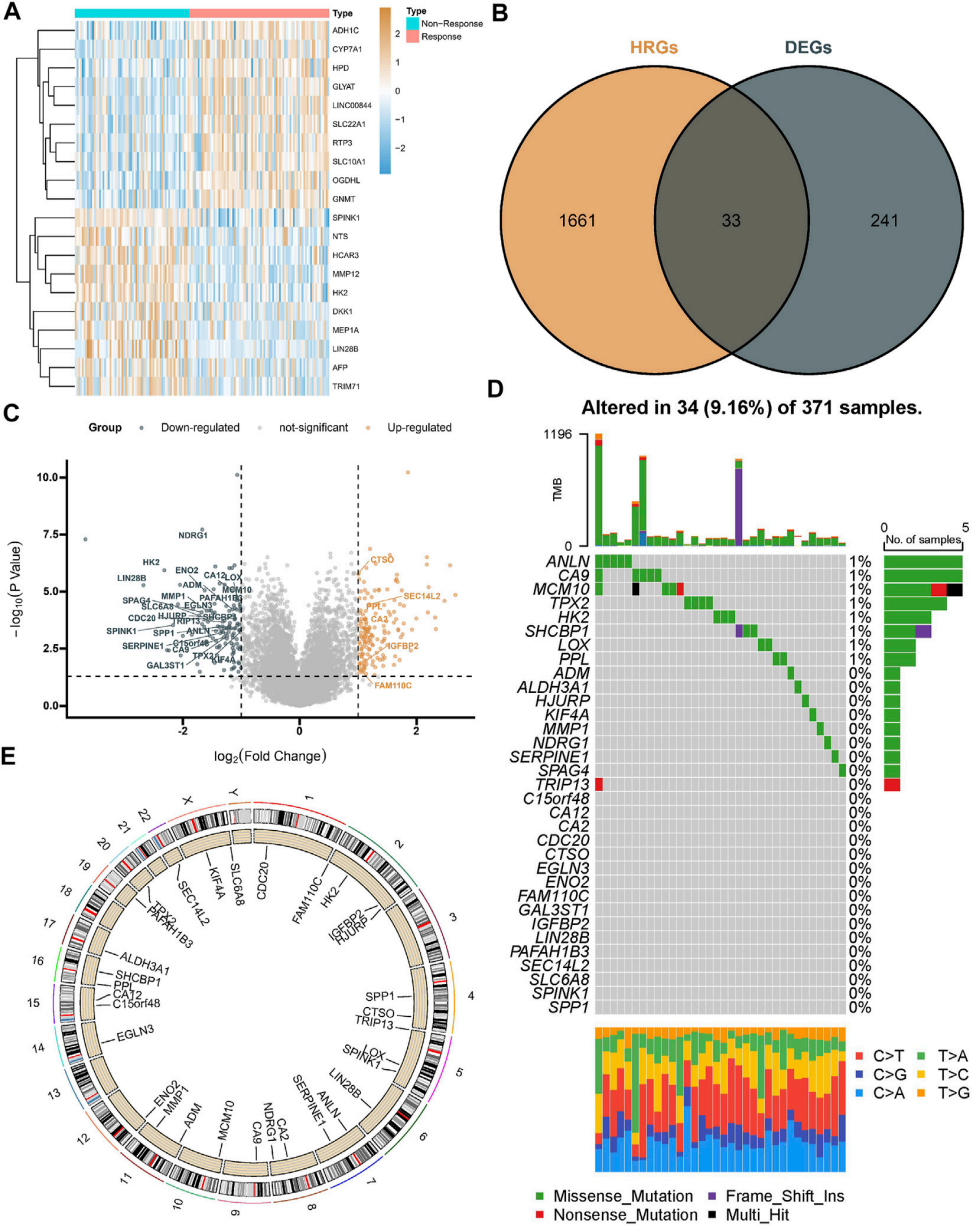
Landscape of HRGTs in HCC. **(A)** The heat map showed a total of DEGs were identified from the GSE104580 cohort. **(B)** The venn plot showed DEGs overlapped with existing HRGs in the database. **(C)** The volcano plot showed dysregulation status of DEGs. **(D)** Mutation landscape of HRGTs in 371 samples. **(E)** The location of HRGTs on the human chromosome.

**FIGURE 2:**
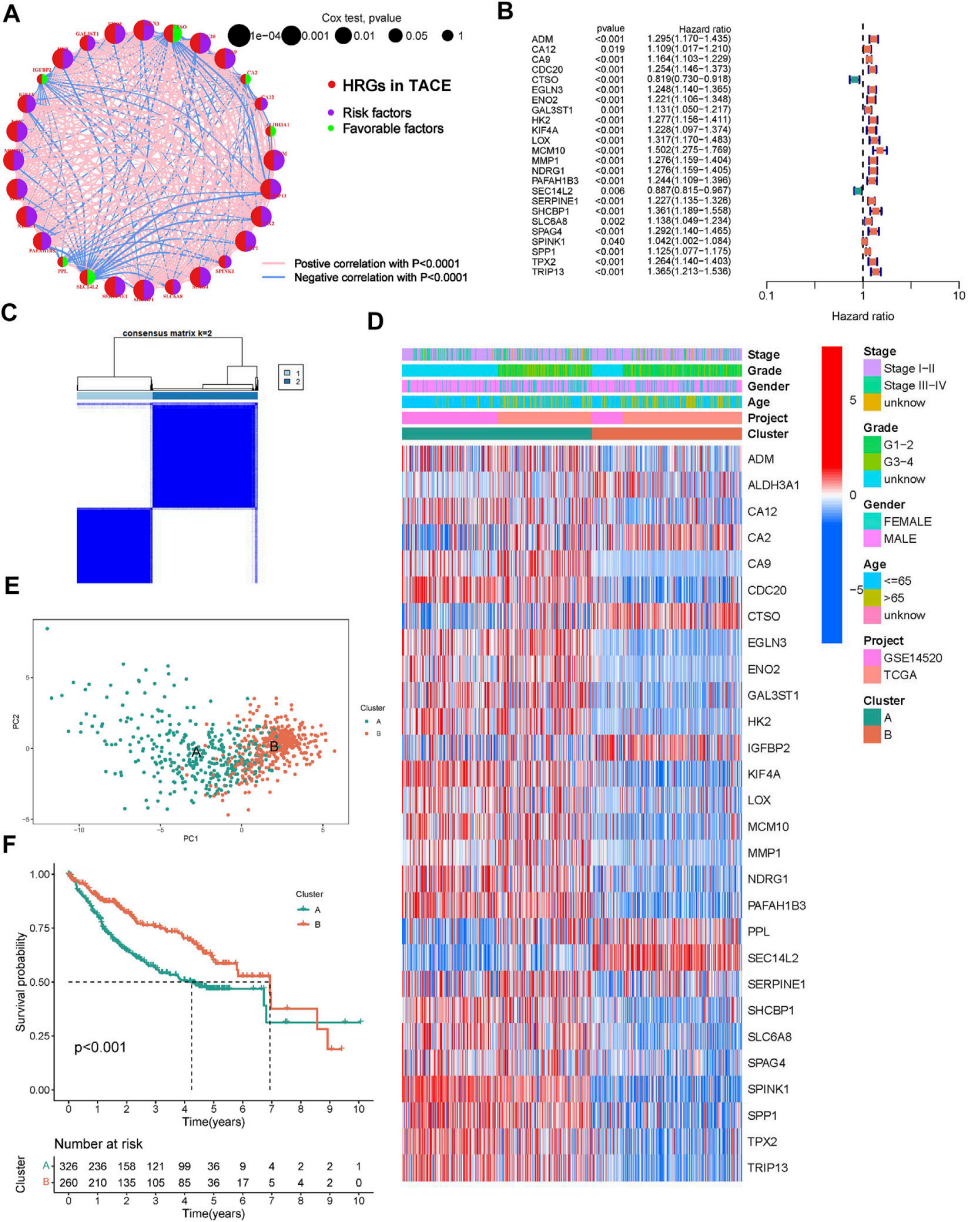
Molecular subtypes based on HRGTs. **(A)** The network of HRGTs described the combined interactions and prognostic significance. **(B)** A forest plot showed 24 HRGTs were indicative of prognostic significance. **(C)** The classification was optimal when the k value = 2. **(D)** Heat map of distribution of clinicopathological characteristics and molecular subtypes based on HRGTs. **(E)** PCA plot revealed that the two molecular subtypes had a relative discrete features. **(F)** Kaplan-Meier analysis of overall survival time in different molecular subtypes.

A total of 274 DEGs were first identified from the GSE104580 cohort (Figures 1A,C) and overlapped with existing HRGs genes in the database. Finally, 33 HRGTs were identified (Figure 1B). The above genes may play a key role in TACE refractoriness. HRGTs were mutated in 34 of 371 samples with a frequency of 9.16%, most of which had a low mutation frequency (Figure 1D). In addition, Copy number variation (CNV) were prevalent in HRGTs. ORG1 focused on copy number amplification, while CNV deletion frequency was common in CDC20 (Supplementary Figure S1A). The location of HRGTs on the chromosome (Figure 1E). GSE14520, TCGA-LIHC were included in a meta cohort using the ‘combat’ algorithm. The network of HRGTs specifically described the combined gene interactions and their prognostic significance for patients (Figure 2A). Cox regression analysis identified 24 HRGTs were indicative of prognostic significance for HCC patients (Figure 2B).

### Identification of molecular subtypes based on HRGTs

The classification was optimal when the k value = 2 (Figure 2C). Two different subtypes were finally identified, with 326 cases in subtype A and 260 cases in subtype B. The heat map showed the distribution of the clinical features of the different subtypes, with most genes significantly overexpressed in subtype A (Figure 2D). PCA plot revealed that the two molecular subtypes had a relative discrete features (Figure 2E). Prognostic analysis revealed a significant survival disadvantage in the subtype B (Figure 2F).

### Immune microenvironment and biological pathways in molecular subtypes

**FIGURE 3:**
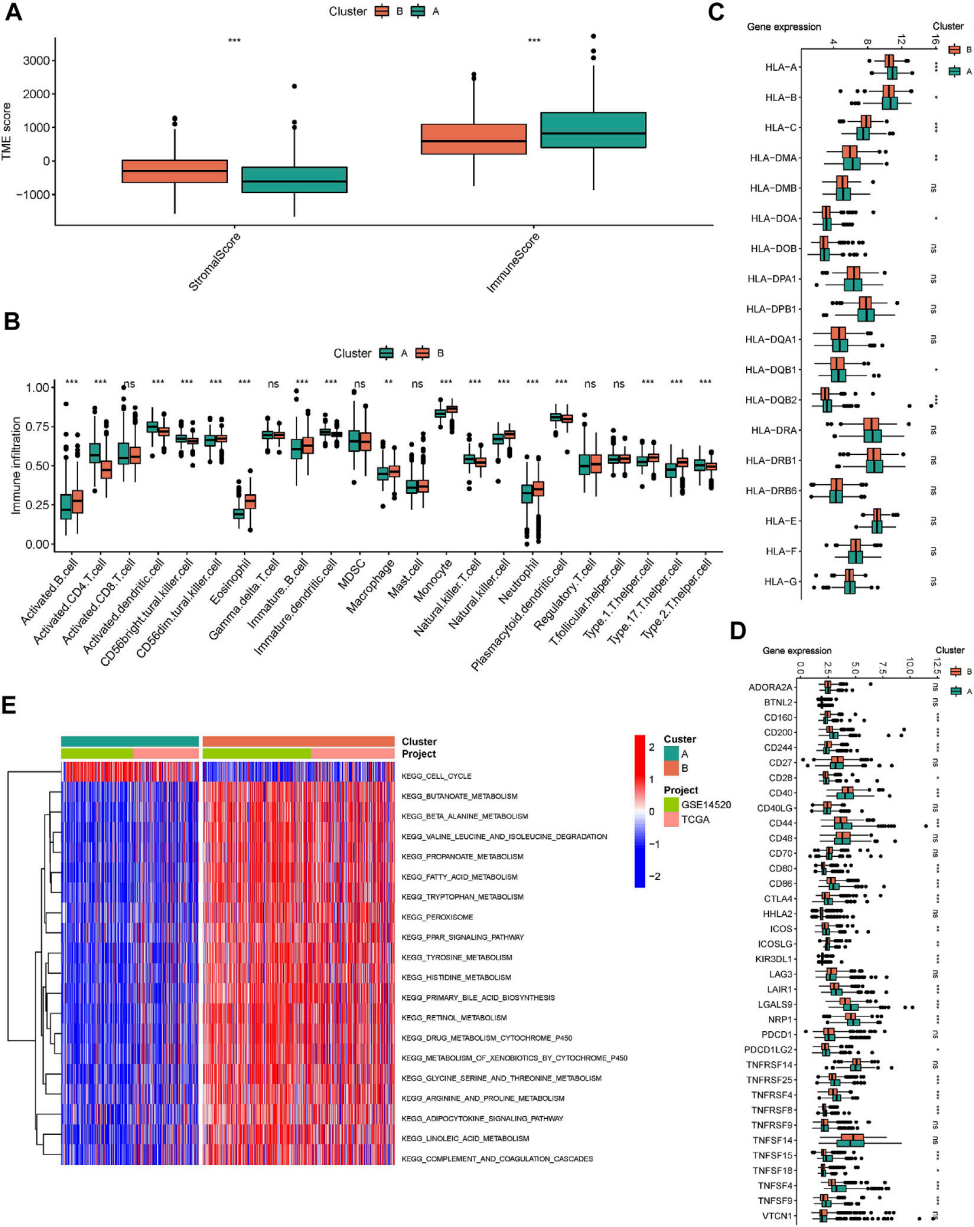
Immune microenvironment and biological pathways in molecular subtypes. **(A)** The Box plot of TME score in different molecular subtypes. **(B)** The Box plot of immune cells score based on ssGSEA in different molecular subtypes. The Box plot of mRNA expression of HLA **(C)** and ICIs **(D)** in different molecular subtypes. **(E)** GSVA analysis in different molecular subtypes using KEGG genesets. *p < 0.05, **p < 0.01, ***p < 0.001.

**FIGURE 4:**
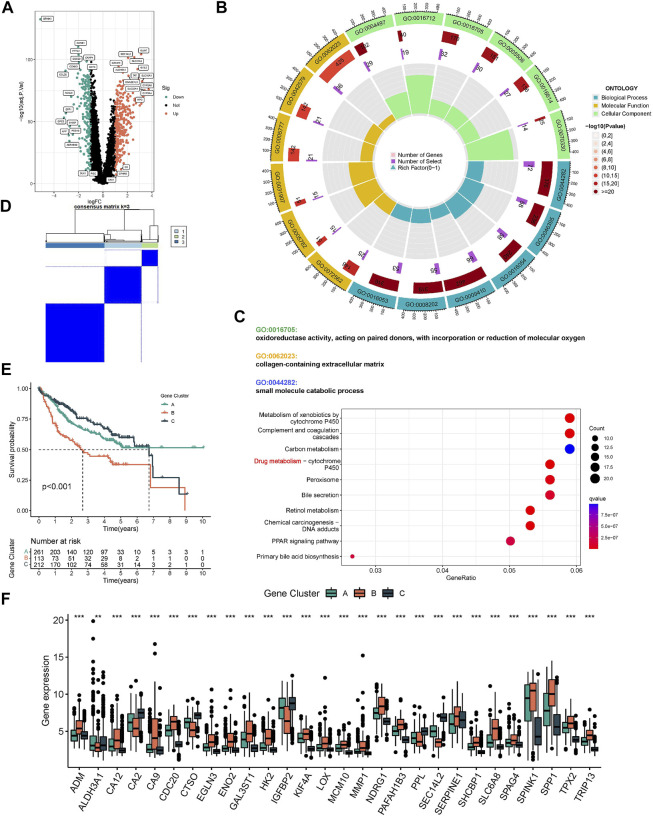
Molecular subtypes based on DEGs. **(A)** The Volcano plot showed DEGs in different subtypes. Heat map of unsupervised clustering analysis. **(B)** The circle plot of GO enrichment analysis based on DEGs. **(C)** The bubble plot of KEGG enrichment analysis based on DEGs. **(D)** The classification was optimal when the k value = 3. **(E)** Kaplan-Meier analysis of overall survival time in different molecular subtypes based on DEGs. **(F)** The Box plot of mRNA expression of HRGTs in different molecular subtypes. *p < 0.05, **p < 0.01, ***p < 0.001.

The ESTIMATE algorithm reveals that subtype A has a higher immune score, while the stromal score was significantly downregulated compared to subtype B (Figure 3A). In addition, ssGSEA analysis demonstrated the TME status in different molecular subtypes. We discovered that subtype A is probably exhibiting hot tumor characteristics. This was due to a significant rise in activated CD4^+^ T cells, which may have a more active TME (Figure 3B). In addition, we showed the expression of HLA as well as ICI mRNA in different subtypes. Interestingly, the subtype A had higher mRNA expression in most HLAs, such as HLA-A, HLA-B, HLA-C, and HLA-DDA (Figure 3C). Similarly, subtype A had higher mRNA expression in most ICIs, such as CTLA4 (Figure 3D). We made a hypothesis that subtype A would benefit more from immunotherapy. To explore the biological behavior between these different subtypes, we performed Gene set variation analysis. Subtype A showed significant enrichment with cell cycle pathways compared to subtype B (Figure 3E). In addition, we performed a differential analysis between the two subtypes. It was found that the major enrichment pathways of the 496 DEGs identified (Figure 4A) may be associated with biological processes related to oxidative stress, extracellular genes and drug metabolism (Figures 4B,C). Finally, we identified three different regulatory subtypes based on the above DEGs (Figure 4D). Among them, subtype B had the worst prognosis, while subtype C had the best prognosis (Figure 4E). And the above 33 HRGTs were differentially expressed in different subtypes (Figure 4F).

### Identification of risk score in HCC

**FIGURE 5:**
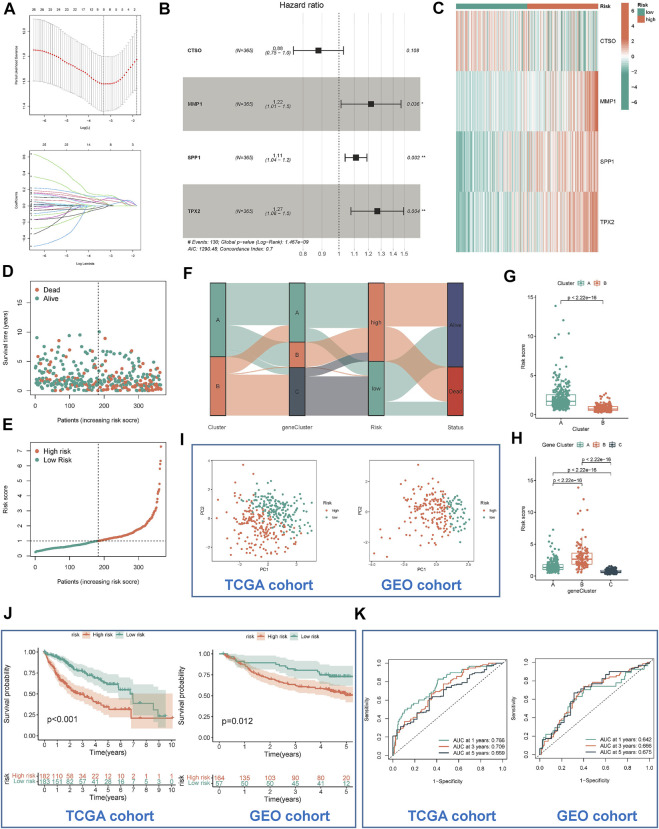
Identification and validation of risk model. **(A)** Determination of the number of regulators using LASSO analysis. **(B)** Forest plot of multivariate Cox regression analysis. **(C)** The heat map showed four HRGTs expression in different risk group (TCGA cohort). Risk status plot **(D)** and the survival distribution plot **(E)** demonstrated the poorer prognosis of HCC patients with higher risk score. **(F)** Sankey diagram based on different subtypes. **(G)** Differences in risk scores between the two molecular subtypes based on HRGTs. **(H)** Differences in risk scores between the three molecular subtypes based on DEGs. PCA plot **(I)**, Kaplan-Meier analysis **(J)**, ROC curve of 1,3,5 years **(K)** of different risk groups in the TCGA and GEO cohort. *p < 0.05, **p < 0.01, ***p < 0.001.

TCGA-LIHC was used as a training cohort with overall survival (OS) as the outcome. The LASSO model was used to remove redundant genes (Figures 5A,B). The coefficients of each gene were obtained by multifactorial Cox regression analysis. A final signature containing 4 HRGTs was obtained. The formula of each patient was as follows: riskscore = (-0.1277 × expression level of *CTSO*) + (0.1995 × expression level of *MMP1*) + (0.1061 × expression level of *SPP1*) + (0.2385 × expression level of *TPX2*). Using the median value of risk scores in the TCGA cohort, we identified two risk groups for HCC patients: high-risk group, and low-risk group in all cohorts. Where *MMP1*, *SPP1*, and TPX2 were significantly highly expressed in the high-risk group, while *CTSO* was significantly highly expressed in the low-risk group (Figure 5C). Among them, the risk status plot and the survival distribution plot demonstrated the poorer prognosis of HCC patients with higher risk score (Figures 5D,E). The results of the sankey plot showed a strong association between risk subtypes and molecular subtypes. And most patients in the subtype A and low risk group were in alive status (Figures 5F–H). PCA also showed genomic heterogeneity between the two risk groups (Figure 5I). Survival analysis and ROC curves indicated (Figures 5J,K) that risk score had a good prognostic value in both the TCGA-LIHC cohort and the GSE14520 cohort, and that survival was suboptimal in patients with both high-risk subtypes. Moreover, we performed correlation analysis between hub genes and m6A methylation regulators, and interestingly, it was positively correlated with most of the regulators except IGFBP1, IGFBP2, and IGF2BP1 (Supplementary Figure S2).

### Association of risk subtypes with immune microenvironment

**FIGURE 6:**
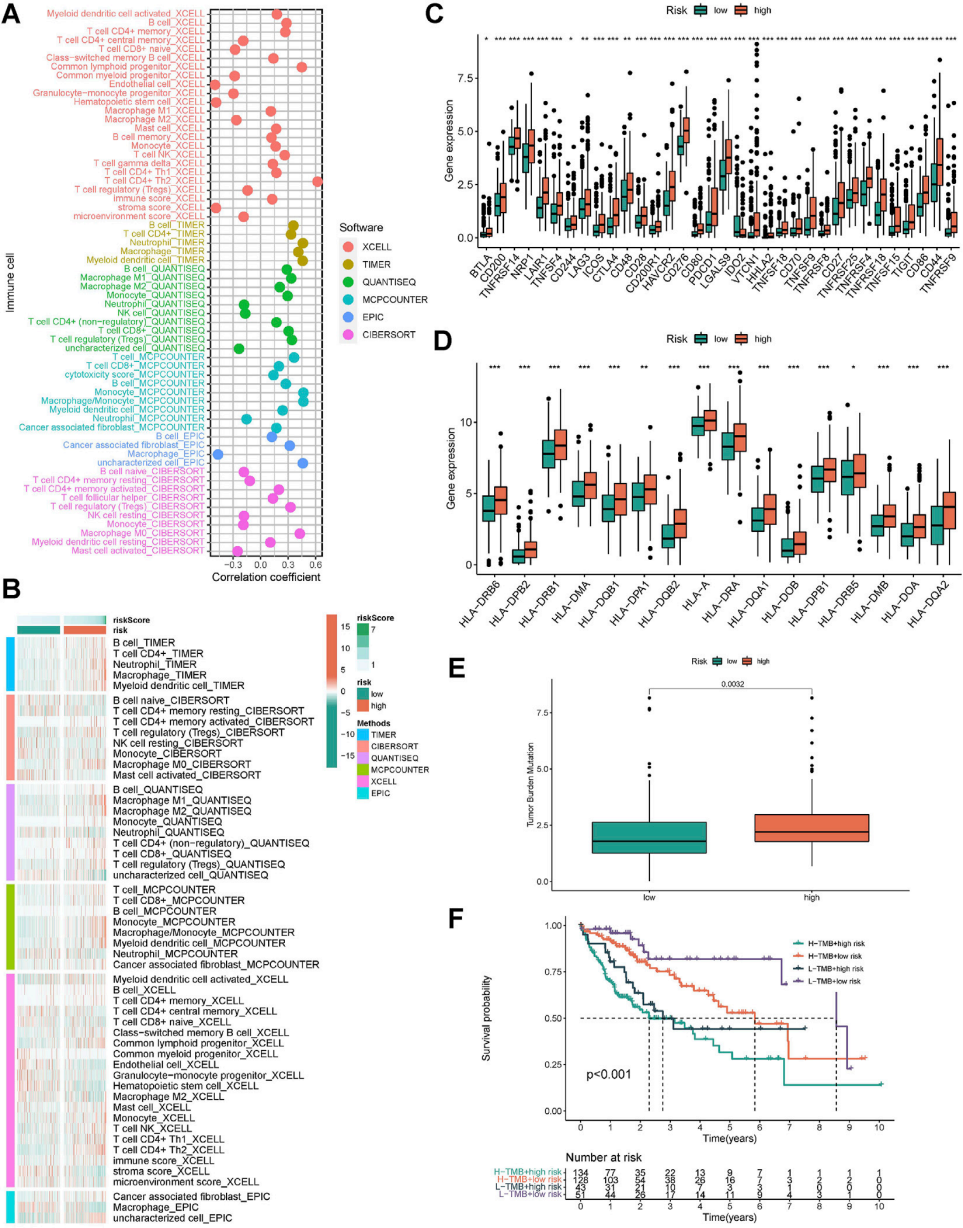
Characteristics of immune microenvironment in different risk groups. **(A)** The heat map showed correlation between risk score and immune function score. **(B)** The heat map showed differences in immune function of different risk groups. **(C)** The box plot showed differences in immune checkpoint mRNA expression of different risk groups. **(D)** The box plot showed differences in HLA mRNA expression of different risk groups. **(E)** The box plot showed differences in TMB score of different risk groups. **(F)** Survival analysis by combining TMB score and risk score. *p < 0.05, **p < 0.01, ***p < 0.001.

We simultaneously applied different algorithms such as TIMER, CIBERSORT, QUANTISEQ, MCP-counter, XCELL and EPIC to estimate the immune cell infiltration status in each samples. Correlation analysis showed that as the risk score increased, the infiltration score of killer immune cells, such as CD4+ T and CD8+ T cell, also increased (Figure 6A). Similarly, there were differences in the distribution of immune cells in the different risk groups. The high-risk group had a more active TME (Figure 6B). In HLA and ICI analysis, the corresponding mRNA expression was higher in high-risk subtypes (Figures 6C,D). Based on whole-exome sequencing data, patients with both high- and low-risk subtypes did not show significant differences in Top mutated genes, which were *TP53*, *CTNNB1*, and *TTN* (Supplementary Figures S1B,C). Considering the importance of tumor mutational burden (TMB) for immunotherapy, we performed a statistical analysis of the TMB differences between the high- and low-risk groups. It was demonstrated that high-risk group had higher TMB, which suggested that they might have a better response to immunotherapy (Figure 6E). Importantly, when low-TMB and low-riskscore are combined, patients will have the best survival advantage (Figure 6F). In addition, we validated our risk signature in immunotherapy cohort (IMvigor210), and the results were consistent with the above findings, namely, high-risk patients had poor survival outcomes, and high-risk patients were more likely to achieve complete remission (CR), as shown in Supplementary Figure S3.

### Risk subtypes could reflect drug resistance in HCC patients

**FIGURE 7:**
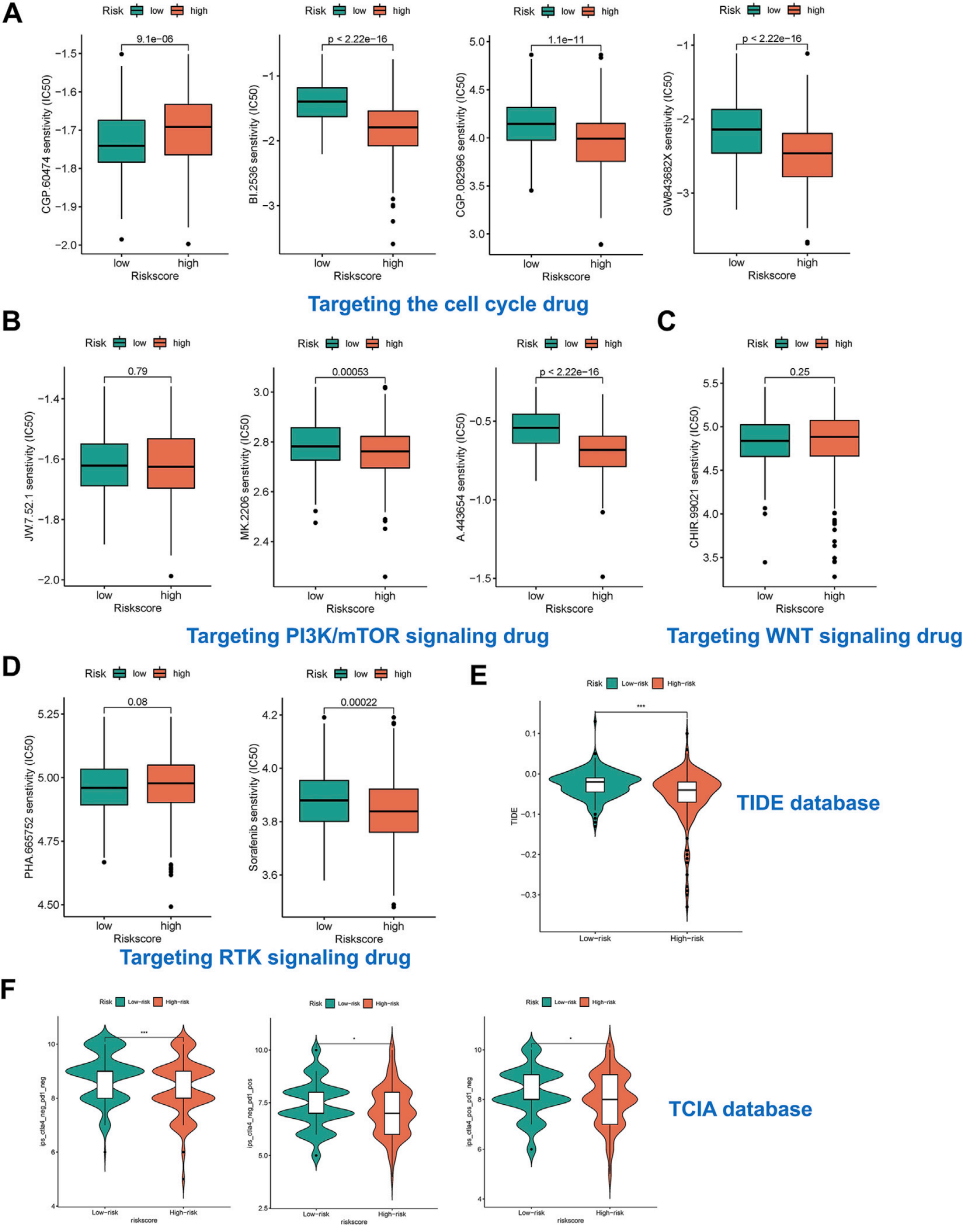
Risk subtypes could reflect drug resistance. **(A)** The box plot of targeting cell cycle drug in different risk groups, including CGP-60474, GW843682x, BI-2536, and CGP-082996. **(B)** The box plot of targeting PI3K/mTOR signaling drug in different risk groups, including JW-7-52-1, MK-2206, and A-443654. **(C)** The box plot of targeting WNT signaling drug in different risk groups, including CHIR-99021. **(D)** The box plot of targeting RTK signaling drug in different risk groups, including Sunitinib and PHA-665752. **(E)** The box plot of TIDE score in different risk groups. **(F)** The box plot of IPS in different risk groups.

We predicted the drug sensitivity of HCC patients in the meta cohort by utilizing the ‘pRRophetic’ algorithm and a ridge regression model. The results showed that targeting cell cycle (CGP-60474, GW843682x, BI-2536, and CGP-082996) (Figure 7A), PI3K/mTOR signaling (JW-7-52-1, MK-2206, and A-443654) (Figure 7B), WNT signaling (CHIR-99021) (Figure 7C), and RTK signaling (Sunitinib and PHA-665752) (Figure 7D) were significantly more effective in high-risk group than in low-risk group. The TIDE score showed that the effectiveness of immunotherapy may be better in high-risk patients (Figure 7E). In addition, the IPS results also demonstrated that the high-risk group seems to have more immunogenic phenotypes (Figure 7F).

### HRGTs in single cell levels

**FIGURE 8:**
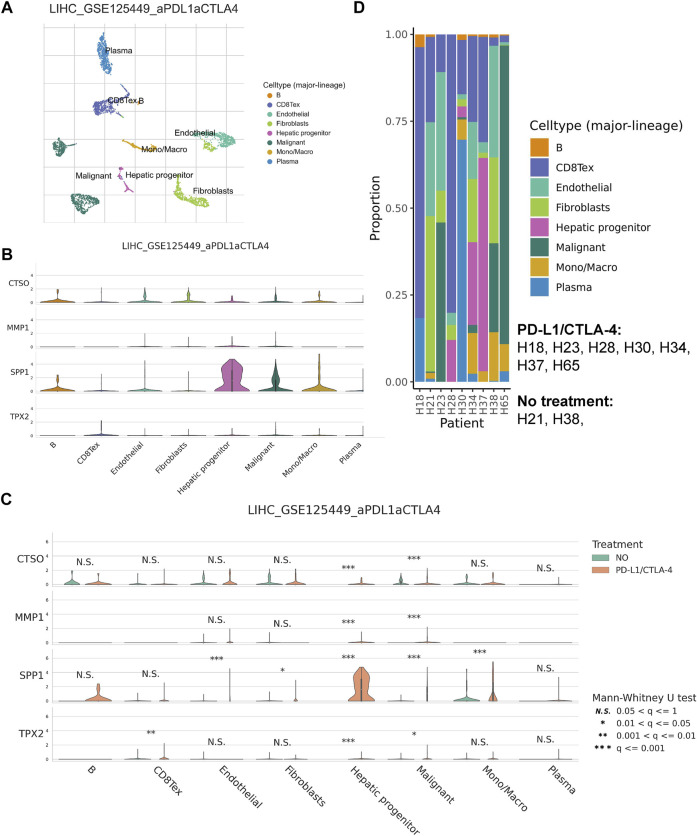
HRGTs in single cell levels. **(A)** t-SNE plot demonstrated the subpopulation of different cells. **(B)** The violin plot demonstrated the expression of *CTSO*, *MMP1*, *SPP1*, and *TPX2* in different cell types. **(C)** The violin plot demonstrated the expression of *CTSO*, *MMP1*, *SPP1*, and *TPX2* in different treatment groups. **(D)** The histogram showed the changes in the proportion of different cell types before and after immunotherapys.

We annotated the GSE125499 single cell expression profile file based on the TISCH database, and t-SNE plot demonstrated the subpopulation of different cells (Figure 8A). In addition, the violin plot demonstrated the expression of *CTSO*, *MMP1*, *SPP1*, and *TPX2* in different cell types, with *SPP1* being more highly expressed in hepatic progenitor (Figure 8B). Interestingly, after PD-L1/CTLA-4 treatment, *CTSO*, *MMP1*, *SPP1*, and *TPX2* were significantly up-regulated in tumor cells (Figure 8C). Finally, we showed the changes in the proportion of different cell types before and after immunotherapy (Figure 8D). The above data suggest to us that four HRGTs involved in risk signature may have a role in reflecting the response to immunotherapy.

## Discussion

Primary liver cancer is one of the sixth most common cancers worldwide and is a common tumor of the digestive system with high aggressiveness and poor prognosis (Choi et al., 2020). Since HCC is not sensitive to conventional radiotherapy, surgery becomes the main treatment method. TACE is the treatment of choice for intermediate-stage hepatocellular carcinoma (Morise et al., 2014). In recent years, however, TACE refractoriness has become a thorny issue and has received increasing attention. This is because TACE accompanied by tumor ischemia plays a dual role in the treatment of HCC. Initially, TACE induces tumor necrosis by blocking the vasculature from the hepatic artery to the HCC. However, TACE also stimulates angiogenesis by inducing hypoxia thereby promoting tumor recurrence and metastasis (Kenji et al., 1997). Tumor angiogenesis and invasiveness by TACE have been found to be mediated by hypoxic signaling, which has been effectively inhibited by antiangiogenic therapies (Liu et al., 2016). However, there are few studies related to HRGs associated with TACE. Although a series of studies have identified predictors or models associated with TACE refractoriness, no studies explore the relevance of HGRTs to the immune microenvironment, prognosis and drug resistance.

Onco-Multi-OMICS approach have been commonly used to discover potential biomarkers (Chakraborty et al., 2018). To date, few studies have constructed prognostic models based on combinations of multiple HRGs in TACE-refractory HCC. Importantly, genetic features and clinical characteristics have performed unsatisfactorily in predicting survival outcomes for TACE-refractory patients. Therefore, we aimed to explore a new HRGTs-based risk stratification and propose potential therapeutic targets for HCC patients. Tumor hypoxia promotes the growth of tumor cells and their transformation to a malignant phenotype. The exploration of hypoxia has opened new perspectives for HCC. Hypoxia is a typical hallmark of TME in almost all solid tumors, caused by rapid and uncontrolled tumor proliferation and inadequate blood supply (Graham and Unger, 2018). Under hypoxic conditions, HIFs bind to transcriptional co-activators and hypoxia response elements to increase the expression of a cascade of target genes, thereby regulating various biological processes, including proliferation, metabolism, angiogenesis, migration and invasion. In addition, hypoxia increases the resistance of tumor cells to chemotherapy, radiotherapy and even immunotherapy (Li et al., 2004). It can inactivate effector cytokine production by inhibiting T cell proliferation and function. Therefore, it is important to fully understand the effects of hypoxia on TACE. In our study, a total of 274 DEGs were first identified in the GSE104580 cohort and overlapped with existing HRGs genes in the database. Finally, identifying 33 HRGTs that may have played a key role in TACE refractoriness. Patients were classified into different subtypes according to the expression of prognostic HRGTs and DEGs. The ESTIMATE algorithm showed that subtype A had a higher immune score, and subtype A had higher mRNA expression of most HLAs and ICIs.

ICIs therapy has been shown to be a highly effective agent for the treatment of HCC(Graham and Unger, 2018). However, it is unclear how to identify those who may benefit most from ICIs therapy. Hypoxia promotes tumor progression in different ways, including proliferation, metabolism, angiogenesis and migration, and improves resistance to ICIs(Bao and Wong, 2021). In addition, many factors, especially in TME, can influence the effectiveness of ICIs(Zhang and Zhang, 2020). The TME is a complex and integral component of cancer, containing tumor cells, stromal cells, inflammatory cells, fibroblasts, metabolites and cytokines. To investigate the value of risk subtypes in TME status and immunotherapy, multiple algorithms were used simultaneously in the immune cell analysis to estimate the immune cell infiltration score in different samples. Correlation analysis showed that as the risk score increased, the infiltration fraction of killer immune cells, such as CD4+ T and CD8+ T cell, also increased (Li et al., 2021). And the high-risk group had more active TME. Corresponding mRNA expression was higher in the high-risk subtype in HLA and ICIs analysis. For drug resistance, our study suggested that the high-risk group may have a better response to immunotherapy. We used the pRRophetic algorithm to predict drug sensitivity of HCC patients in different risk groups. The results showed that drugs targeting cell cycle, PI3K/mTOR signaling, WNT signaling, and RTK signaling were more effective in high-risk patients. Importantly, the IPS results demonstrated that the high-risk group seems to have more immunogenic phenotypes.

For the four HRGTs involved in the risk signature, we found that all of them were associated with tumor immunity. Secretory phosphorylated protein 1 (*SPP1*) is a secreted multifunctional phosphorylated protein that specifically binds and activates matrix metalloproteinases (MMPs) in cancer (Chen et al., 2019a). Its main biological functions are involved in immune response, biomineralization and tissue remodeling. *SPP1* has also been implicated in cell growth, proliferation, migration, apoptosis and chemotaxis. Previous studies have demonstrated that *SPP1* is overexpressed in a variety of cancers and can be used to predict chemotherapy prognosis, such as ovarian cancer (Zeng et al., 2018), glioblastoma (Kijewska et al., 2017), HCC (Liu et al., 2022) and gastric cancer (Chen et al., 2018a). *MMP1* is a member of a family of zinc-dependent endopeptidases involved in wound healing, inflammation, cancer and angiogenic remodeling of the extracellular matrix (ECM) (Chen et al., 2019b). It has been shown to be closely associated with migration and invasion in many cancers. mmp1 promotes cell cycle acceleration in cancer cells by activating the cdc25a/CDK4-cyclin D1 and p21/cdc2-cyclin B1 complexes (Yu et al., 2021). A newly discovered mechanism of *MMP1* in tumor promotion is by activating *PAR1* to cleave downstream oncogenic signaling pathways (Huang et al., 2018). This is expected to be a promising strategy to address the TACE refractoriness. *TPX2* has been identified as an oncogenic factor in a variety of cancers. For example, upregulated expression of *TPX2* enhances breast cancer metastasis by mediating *MMP2* and *MMP9* expression (Tan et al., 2019). In addition, *TPX2* can inhibit cell proliferation and enhance apoptosis by blocking the PI3k/AKT/p21 pathway and activating the p53 pathway in breast cancer (Chen et al., 2018b). It has been shown that *TPX2* is highly expressed in HCC tissues. *TPX2* expression is associated with the infiltration status of immune cells in HCC involving B, CD4^+^T and CD8^+^ T cells, neutrophils, macrophages and DCs(Zhu et al., 2020). In addition, CDK5-mediated stabilization of *TPX2* promotes HCC tumorigenesis (Wang et al., 2019). Clearly, these studies suggest that *TPX2* is an unfavorable marker for HCC and holds promise as a therapeutic target for TACE refractoriness. *CTSO* is a cysteine protease that has been shown to have both extracellular and intracellular functions. This class of proteases mediates intracellular protein catabolism and selectively activates extracellular protein degradation, macrophage function and bone resorption (Shi et al., 1995). The role in cancer therapeutic resistance is an emerging area of interest.

In our study, different hypoxic patterns present different biological processes, signaling pathways and immune features. Based on the parameters of the risk model, we determined that high-risk patients have a more active immune microenvironment, and HRGs are potential biomarkers for TACE-refractory patients. Especially, it may be an independent prognostic factor for HCC patients. However, our study has some limitations. Firstly, we should use advanced artificial intelligence models rather than traditional machine learning models such as Random Forest (RF) or LASSO models. However, for clinical applications, machine learning models with coefficients may be more helpful to clinicians. The clinician can calculate the survival risk of each patient from the mRNA expression and coefficient, however, more advanced deep learning models are a ‘black box’. Moreover, due to the limitation of laboratory conditions, we have no more time to conduct *in vivo* or vitro experiments, and we will validate the mechanism of four hub genes in TACE-refractory patients in the future. In conclusion, our study will provide a novel immunological perspective for the development of treatment options for TACE-refractory HCC.

## Data Availability

The datasets presented in this study can be found in online repositories. The names of the repository/repositories and accession number(s) can be found in the article/Supplementary Material.

## Supplementary material

Supplementary Table S1 Treatment details for HCC patients in GSE14520.
