# Supplementary material for: The role of hypoxia-related genes in TACE-refractory hepatocellular carcinoma: Exploration of prognosis, immunological characteristics and drug resistance based on onco-multi-OMICS approach
Source: Front Pharmacol. 2022 Sep 26;13:1011033. doi: 10.3389/fphar.2022.1011033 (PMC9549174; doi:10.3389/fphar.2022.1011033)
Supplement: Supplementary file 1 [file Table1.DOCX]

| Adjuvant TACE subset | Post-Recurrence TACE subset | Resection Only | Other Therapy |
| --- | --- | --- | --- |
| LCS_007A | LCS_008A | LCS_010A | LCS_002A |
| LCS_009A | LCS_012A | LCS_014A | LCS_004A |
| LCS_019A | LCS_023A | LCS_015A | LCS_005A |
| LCS_020A | LCS_024A | LCS_016A | LCS_011A |
| LCS_025A | LCS_032A | LCS_018A | LCS_021A |
| LCS_027A | LCS_035A | LCS_022A | LCS_036A |
| LCS_028A | LCS_067A | LCS_040A | LCS_039A |
| LCS_029A | LCS_072A | LCS_041A | LCS_042A |
| LCS_031A | LCS_088A | LCS_044A | LCS_054A |
| LCS_033A | LCS_096A | LCS_045A | LCS_066A |
| LCS_034A | LCS_120A | LCS_046A | LCS_074A |
| LCS_038A | LCS_138A | LCS_048A | LCS_083A |
| LCS_043A | LCS_139A | LCS_051A | LCS_089A |
| LCS_047A | LCS_145A | LCS_056A | LCS_093A |
| LCS_049A | LCS_178A | LCS_057A | LCS_095A |
| LCS_050A | LCS_190A | LCS_061A | LCS_103A |
| LCS_062A | LCS_194A | LCS_063A | LCS_107A |
| LCS_065A | LCS_198A | LCS_064A | LCS_123A |
| LCS_068A | LCS_200A | LCS_069A | LCS_125A |
| LCS_071A | LCS_207A | LCS_073A | LCS_126A |
| LCS_075A | LCS_224A | LCS_076A | LCS_129A |
| LCS_079A | LCS_227A | LCS_078A | LCS_135A |
| LCS_085A | LCS_234A | LCS_084A | LCS_143A |
| LCS_086A | LCS_238A | LCS_090A | LCS_148A |
| LCS_092A | LCS_267A | LCS_091A | LCS_149A |
| LCS_097A | LCS_273A | LCS_094A | LCS_152A |
| LCS_100A | LCS_274A | LCS_099A | LCS_153A |
| LCS_104A | LCS_281A | LCS_101A | LCS_157A |
| LCS_110A | LCS_333A | LCS_102A | LCS_162A |
| LCS_116A | LCS_403A | LCS_105A | LCS_164A |
| LCS_117A |  | LCS_106A | LCS_173A |
| LCS_118A |  | LCS_108A | LCS_175A |
| LCS_121A |  | LCS_109A | LCS_182A |
| LCS_127A |  | LCS_119A | LCS_183A |
| LCS_134A |  | LCS_122A | LCS_188A |
| LCS_136A |  | LCS_130A | LCS_193A |
| LCS_140A |  | LCS_131A | LCS_195A |
| LCS_142A |  | LCS_132A | LCS_199A |
| LCS_146A |  | LCS_137A | LCS_201A |
| LCS_154A |  | LCS_144A | LCS_203A |
| LCS_158A |  | LCS_147A | LCS_206A |
| LCS_159A |  | LCS_150A | LCS_219A |
| LCS_161A |  | LCS_151A | LCS_230A |
| LCS_166A |  | LCS_156A | LCS_248A |
| LCS_167A |  | LCS_160A | LCS_250A |
| LCS_170A |  | LCS_163A | LCS_256A |
| LCS_171A |  | LCS_165A | LCS_277A |
| LCS_177A |  | LCS_169A | LCS_290A |
| LCS_185A |  | LCS_172A | LCS_339A |
| LCS_191A |  | LCS_174A | LCS_341A |
| LCS_192A |  | LCS_179A | LCS_401A |
| LCS_196A |  | LCS_180A |  |
| LCS_197A |  | LCS_184A |  |
| LCS_208A |  | LCS_189A |  |
| LCS_209A |  | LCS_205A |  |
| LCS_212A |  | LCS_210A |  |
| LCS_213A |  | LCS_211A |  |
| LCS_223A |  | LCS_215A |  |
| LCS_228A |  | LCS_216A |  |
| LCS_231A |  | LCS_222A |  |
| LCS_240A |  | LCS_236A |  |
| LCS_241A |  | LCS_237A |  |
| LCS_245A |  | LCS_243A |  |
| LCS_251A |  | LCS_247A |  |
| LCS_259A |  | LCS_249A |  |
| LCS_260A |  | LCS_253A |  |
| LCS_263A |  | LCS_254A |  |
| LCS_264A |  | LCS_261A |  |
| LCS_265A |  | LCS_262A |  |
| LCS_266A |  | LCS_268A |  |
| LCS_270A |  | LCS_269A |  |
| LCS_272A |  | LCS_275A |  |
| LCS_284A |  | LCS_278A |  |
| LCS_289A |  | LCS_279A |  |
| LCS_393A |  | LCS_282A |  |
|  |  | LCS_285A |  |
|  |  | LCS_286A |  |
|  |  | LCS_291A |  |
|  |  | LCS_343A |  |
|  |  | LCS_344A |  |
|  |  | LCS_346A |  |
|  |  | LCS_400A |  |
|  |  | LCS_406A |  |
|  |  | LCS_415A |  |
|  |  | LCS_424A |  |
|  |  | LCS_426A |  |
