# Supplementary figures and images for: The role of hypoxia-related genes in TACE-refractory hepatocellular carcinoma: Exploration of prognosis, immunological characteristics and drug resistance based on onco-multi-OMICS approach
Source: Front Pharmacol. 2022 Sep 26;13:1011033. doi: 10.3389/fphar.2022.1011033 (PMC9549174; doi:10.3389/fphar.2022.1011033)

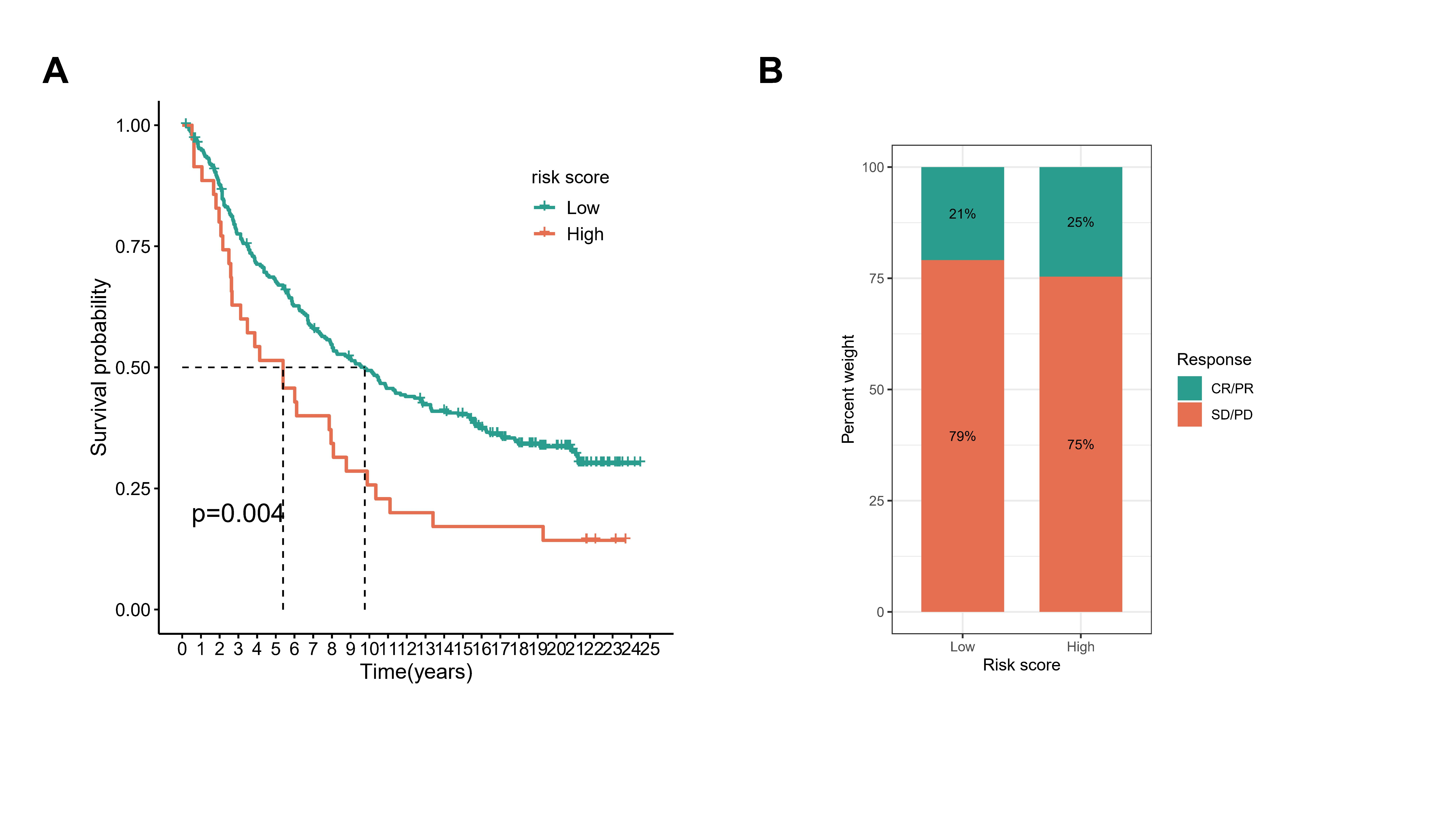

Supplement: Supplementary file 2 [file Image3.TIF]

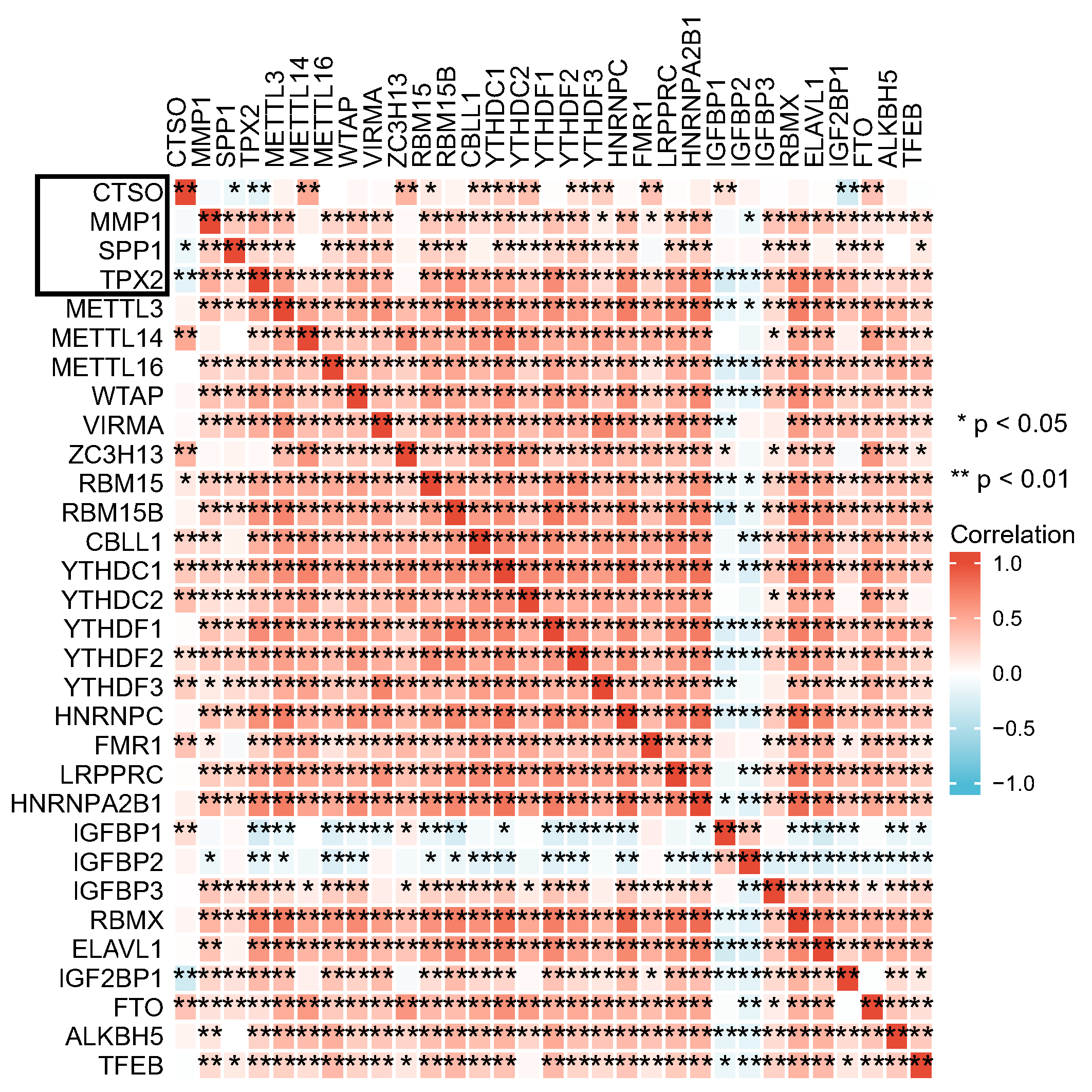

Supplement: Supplementary file 3 [file Image2.TIF]

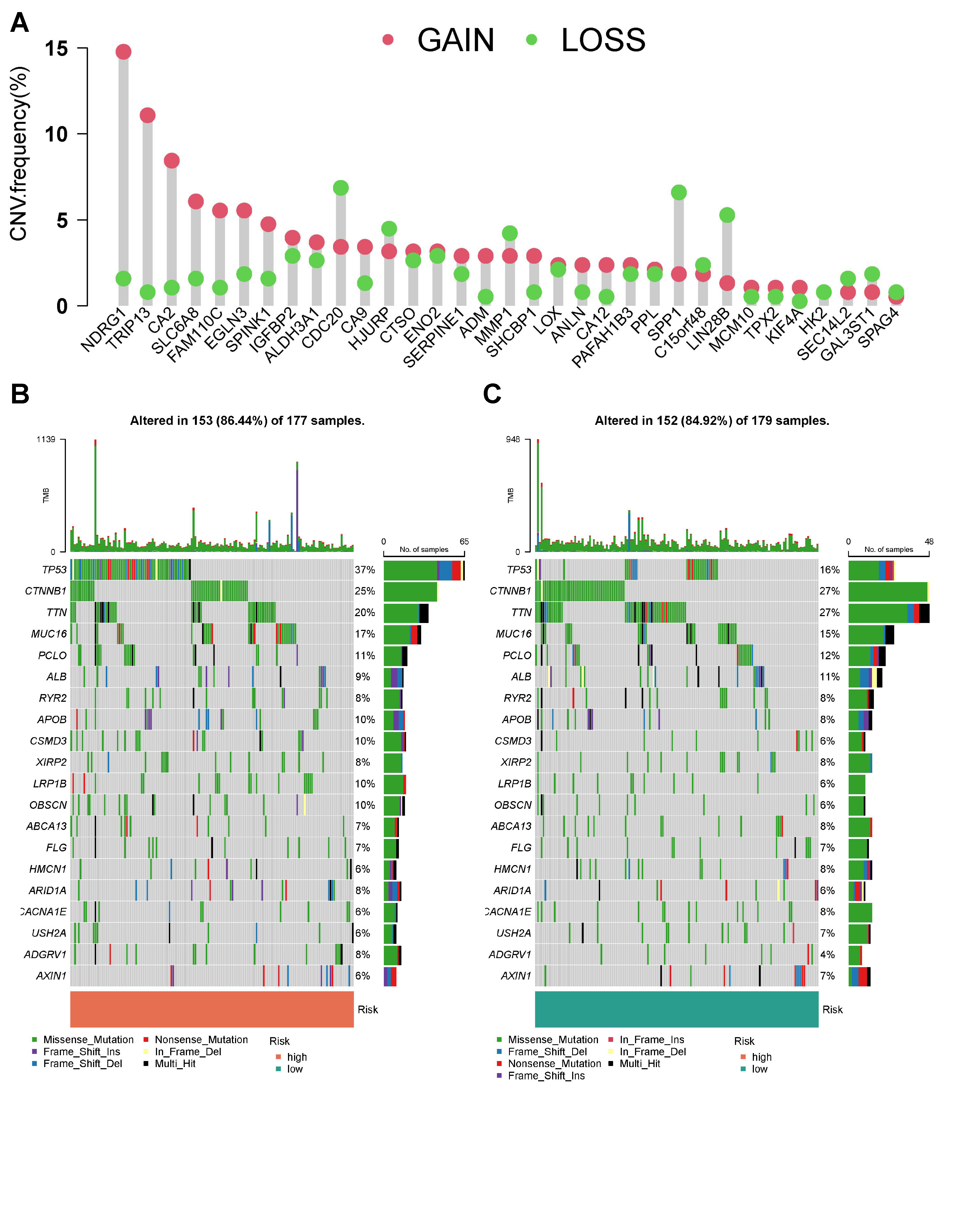

Supplement: Supplementary file 4 [file Image1.TIF]
